# Architecture and Florogenesis in Female *Cannabis sativa* Plants

**DOI:** 10.3389/fpls.2019.00350

**Published:** 2019-04-02

**Authors:** Ben Spitzer-Rimon, Shai Duchin, Nirit Bernstein, Rina Kamenetsky

**Affiliations:** ^1^ Institute of Plant Sciences, Agricultural Research Organization, The Volcani Center, Rishon LeZion, Israel; ^2^ Institute of Soil Water and Environmental Sciences, Agricultural Research Organization, The Volcani Center, Rishon LeZion, Israel

**Keywords:** cannabis, inflorescence, photoperiod, solitary flower, branching

## Abstract

The inflorescence is the main product of medical cannabis. Hundreds of specialized metabolites with potential bioactivity are produced and accumulated in the glandular trichomes that are highly abundant mainly on female inflorescences. Understanding the morphophysiological and genetic mechanisms governing flower and inflorescence development is therefore of high scientific and practical importance. However, in-depth investigations of cannabis florogenesis are limited. Cannabis producers and researchers consider long photoperiod to be “non-inductive” or “vegetative,” but under these growth conditions, the development of solitary flowers and bracts in shoot internodes clearly indicates that the plant cannot be defined as vegetative or non-inductive in the classical sense. Most probably, induction of solitary flowers is age-dependent and controlled by internal signals, but not by photoperiod. Short photoperiod induces intense branching, which results in the development of a compound raceme. Each inflorescence consists of condensed branchlets with the same phytomer structure as that of the larger phytomers developed under long day. Each phytomer consists of reduced leaves, bracts, one or two solitary flowers, and an axillary shoot (or inflorescence). Therefore, the effect of short photoperiod on cannabis florogenesis is not flower induction, but rather a dramatic change in shoot apex architecture to form a compound racemose inflorescence structure. An understanding of the morphophysiological characteristics of cannabis inflorescence will lay the foundation for biotechnological and physiological applications to modify architecture and to maximize plant productivity and uniformity in medical *Cannabis*.

## Introduction

The genus *Cannabis*, in the family Cannabaceae, includes annual herbaceous, dioecious species. For a long time, the taxonomic status of the genus was inconclusive, and the number of *Cannabis* species is still controversial (Small et al., 1976; Hillig, 2005; Chandra et al., 2017; Small, 2017; McPartland, 2018). The most commonly agreed upon formal taxonomy for this plant is that the genus *Cannabis* comprises one species, *C. sativa* L., with highly polymorphic subspecies *sativa*, *indica*, and *ruderalis.* These subspecies differ in their phenotypic characteristics and chemical profiles (Small et al., 1976; Small, 2015; McPartland, 2018; Zhang et al., 2018). *Cannabis* is most probably indigenous to and originating from Central Asia and upper southern Asia (Clarke and Merlin, 2013). Intensive crossbreeding between subspecies resulted in the elimination of each population’s differences and unique characteristics, and determining the origin of modern cultivars has become a challenge (McPartland, 2018). On the other hand, *Cannabis* interbreeding has contributed to the enormous phenotypic and chemical diversity of *Cannabis* cultivars that are in use today (Hillig and Mahlberg, 2004; Andre et al., 2016; Hazekamp et al., 2016).

**Figure 1:**
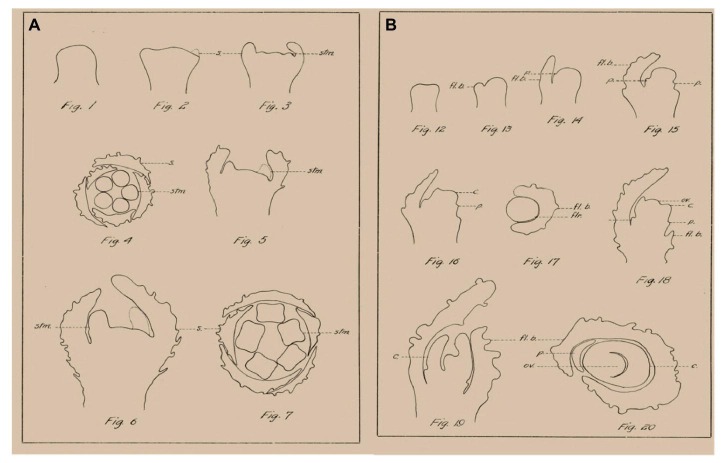
Examples of morphological analysis of *Cannabis* flowers by Camera Lucida, adapted from Reed (1914). **(A)** Morphogenesis of staminate flower. **(B)** Morphogenesis of pistillate flower. s., sepal; stm., stamen; fl. b., floral bract; p., perianth; c., carpel; flr., flower; ov., ovule.

*Cannabis* contains hundreds of specialized metabolites with potential bioactivity, including cannabinoids, terpenes, and flavonoids, which are produced and accumulated in the glandular trichomes that are highly abundant mainly on female inflorescences (Hammond and Mahlberg, 1977; Andre et al., 2016; Chandra et al., 2017; Raman et al., 2017). Since this complex specialized metabolite profile defines the medical and commercial potential of cannabis, the female inflorescence has attracted much attention (Small, 2016; Chandra et al., 2017; Grof, 2018). *Cannabis* cultivars used for medical purposes are considered to have a short photoperiod requirement for flowering. Since the inflorescence is the main product of medical cannabis, understanding the morphophysiological and genetic mechanisms of flower and inflorescence development is of high scientific and practical importance. However, in-depth investigations of cannabis florogenesis are limited. One of the first detailed morphological descriptions of cannabis floral organs and their development was described in 1914 by Joyce Reed, and the figures in that paper, by Camera Lucida, provide some interesting and useful information (Figure 1; Reed, 1914). In the last century, knowledge on florogenesis and its genetic regulation has greatly increased, and inflorescence typology and terminology have changed. With the easing of legal restrictions concerning cannabis research, new scientific tools can now be applied for reevaluation and in-depth studies of florogenesis and flowering control in cannabis.

In general, plant inflorescences are branches that bear flowers. Following a vegetative phase, there is a transition to the reproductive phase, and the shoot apical meristem (SAM) is transformed into an inflorescence meristem. The latter can produce axillary meristems that develop into inflorescences of higher order or into individual flowers. The inflorescence meristem is thus a transient stage between two main types of meristems: vegetative meristem, which produces leaves and stems, and floral meristem, which terminates by producing the reproductive organs (Benlloch et al., 2007; Prenner et al., 2009; Castel et al., 2010; Park et al., 2014). Branching of the inflorescence follows regular patterns. As a rule, a new branch is formed in the axil of a foliage leaf or a bract. This leaf is called the subtending leaf or pherophyll of the new branch. Pherophylls are not restricted to inflorescences but are of general occurrence in a ramifying flowering plant. In inflorescences, pherophylls are more often bracts than foliage leaves. However, not every bract must have a flower in its axil, because an initiated axillary bud may not develop further (Endress, 2010). There are a number of basic types of inflorescences, including cymose or racemose inflorescence, panicle, and thyrse (Benlloch et al., 2007; Prenner et al., 2009; Castel et al., 2010). Branching patterns in racemose and cymose inflorescences are contrast. In the racemose pattern, the main axis produces numerous lateral branches of the second order. The main axis can be terminated by a flower (determinate inflorescence) or not (indeterminate inflorescence). In contrast, in the cymose inflorescence, the main axis has no more than two second-order branches and no more than two extrafloral leaves (phyllomes). The number of branching orders is not limited. In a cymose pattern, the main axis is commonly terminated by a flower. In both racemose and cymose patterns, the plant can produce variable number of flowers per inflorescence. Two additional inflorescence types, thyrse and panicle, are intermediate between cymose and racemose patterns (Endress, 2010).

Terminology for phenological stages of *Cannabis* development and flowering has been recently proposed by several authors (Farag and Kayser, 2017; Mishchenko et al., 2017; Raman et al., 2017). In horticultural practice, *Cannabis* is propagated by rooted cuttings, with two bracts and a solitary flower primordium developing in the axil of each stipulate leaf (Cervantes, 2006; Caplan et al., 2018). Development of these solitary flowers is the first visual indication of the plant’s sex, and in horticultural practice, they are used to discriminate between female and male plants at relatively early developmental stages.

While the nomenclature of female flowers is abundantly presented on non-scientific websites^1^, the flowering terminology is often controversial and confusing. Therefore, the present study focused on a morphophysiological analysis of female cannabis plants. Plant architecture and timing of initiation and differentiation of the inflorescence and individual flowers of three cultivars are described and illustrated. This research provides a basis for further molecular genetic investigations of the cannabis flowering system.

## Materials and Methods

### Plant Material and Growth Conditions

Three medical cultivars of *Cannabis sativa* L., NB130, NB140, and NB150 (Canndoc Ltd., Israel), were used as model systems in this study. “NB130” is a ~7%/7% Δ^9^-tetrahydrocannabinol (THC)/cannabidiol (CBD) cultivar with *sativa* dominant phenotype; “NB140” and “NB150” are high THC cultivars (~15%/0.03% THC/CBD) with *indica* dominant phenotype and *sativa-indica* mixed phenotype, respectively. The plants were propagated from cuttings of a single female mother plant in a coconut fiber mixture. Rooted cuttings were transferred to 200-ml pots for 14 days and then transferred to 2-L plastic pots, one cutting per pot, in a coconut/perlite growing mixture (Tuff Merom Golan, Israel) and cultivated in a controlled environment for an additional 1 week under long photoperiod (16/8 h light/dark), which is referred to in the literature and by cannabis growers as vegetative growth conditions. MH bulbs (1,000 W) provided a light intensity of 600 μmol m^−2^ s^−1^ (GrowLite Tru Blue, GrowLite Inc., Glendale, AZ, USA). Thereafter, the plants were transferred to a short (12/12 h) photoperiod under 1000 W HPS bulbs (Grow lite Real Red HPS) with a light intensity of 1,000 μmol m^−2^ s^−1^. Light intensity was confirmed using an Apogee MQ-500 PAR meter (Apogee Instruments, Logan, UT, USA). Temperature in the growth room was 25°C, and relative humidity was 40 and 60% day/night, respectively. Temperature and humidity were continuously recorded using an EC850A MicroLog Pro (Fourtec-Fourier Technologies, Orland Park, IL, USA). Irrigation was supplied *via* 1 L h^−1^ discharge-regulated drippers (Plastro-Gvat, Kibbutz Gvat, Israel), 1 dripper per pot (Bernstein et al., 2019). The volume of irrigation was 500–800 ml/pot/day, set to allow 35–40% of drainage. Fertilizers were supplied by fertigation, i.e., dissolved in the irrigation solution at each irrigation event in the concentration of 85 ppm N (with 1:2 ratio of NH_4_^+^/NO_3_^−^), 40 ppm P_2_O_5_ (17 ppm P), and 108 ppm K_2_O (90 ppm K). Micronutrients were supplied chelated with EDTA in the concentrations of 0.4 ppm Fe, 0.2 ppm Mn, and 0.06 ppm Zn. On each sampling date, three individual healthy plants and/or apical and lateral meristems were randomly picked for macro- and micromorphogenetic analyses.

### Microscopy

Plants of each cultivar were sampled for meristem analysis every 5–7 days. Analyses were conducted with three replicate plants per cultivar on each sampling day. Sampled plants were carefully stripped of their leaves, and leaves were also removed from the developing floral buds. Isolation of meristems or developing inflorescences was performed under a stereomicroscope (Olympus model SZX10, Japan).

For scanning electron microscopy (SEM), the excised meristems were fixed in ethanol (70%) and dehydrated in a graded ethanol series (90 and 100%). Tissues were then immediately dried using liquid CO_2_ in a K-850 critical point dryer (Quorum Technologies, Laughton, UK). Samples were mounted on SEM stubs with double-sided tape, sputter-coated with about 10 nm of palladium in a SC7620 mini sputter coater (Quorum Technologies), and studied in a Jeol JCM-6000 scanning electron microscope (Akishima, Japan) with an accelerating potential of 15 kV.

## Results

### Growth and Development Under Long Photoperiod

**Figure 2:**
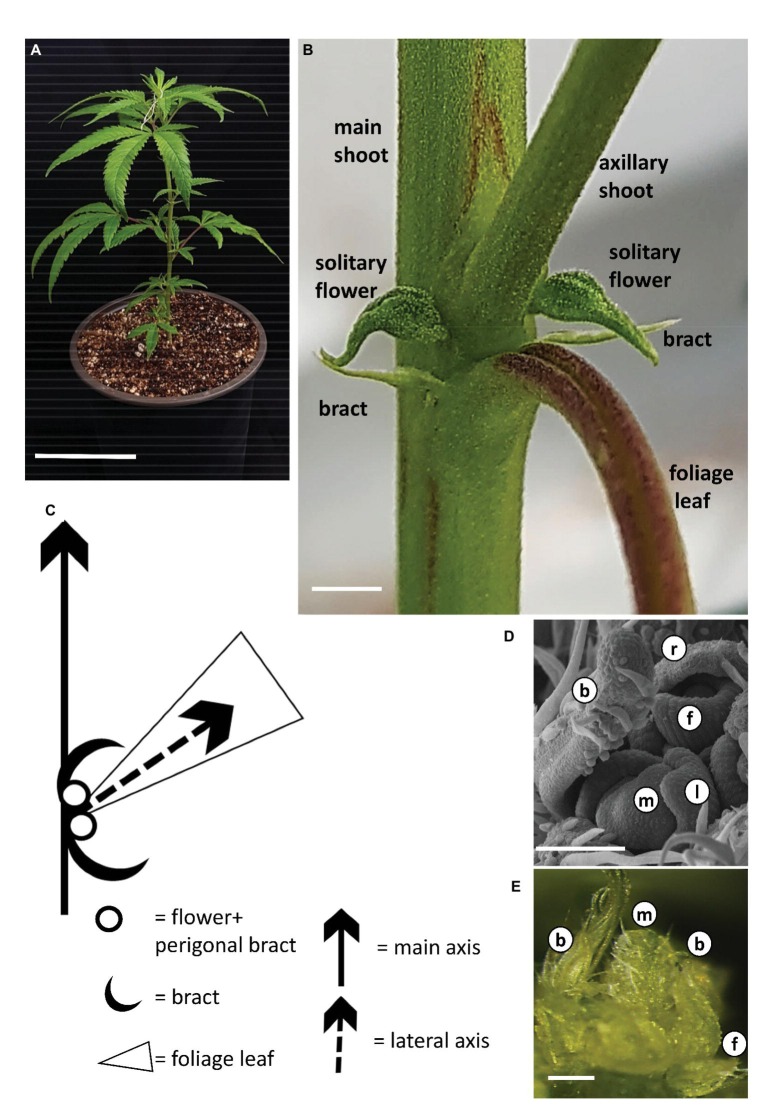
Growth and development of *Cannabis* under long photoperiod. **(A)** Young rooted cutting of cv. NB140 2 weeks after rooting. Bar = 10 cm. **(B)** Internode of cannabis plant. Axillary shoot, two bracts, and two solitary flowers are located in the axil of a foliage leaf. Bar = 0.2 cm. **(C)** Schematic representation of the basic phytomer, including internode, foliage (fan) leaf, two bracts, and two solitary flowers. **(D)** Scanning electron photomicrograph of cannabis apical meristem. Bar = 100 μm. (**E)** Stereoscope image of cannabis apical meristem producing leaves, solitary flowers, and bracts. External leaves removed to expose the meristem. Bar = 200 μm. b, bract primordium; f, flower primordium; l, leaf primordium; m, meristem; r, perigonal bract.

During growth under long photoperiod, the main shoot of the cannabis plants branched monopodially, producing alternate branching shoots (Figure 2A). The monopodial plant consisted of numerous phytomers, each of which included an internode with one large photosynthetic palmately compound leaf (foliage leaf or fan leaf) and axillary shoot secondary phytomer. Two bracts were located on each side of the leaf petiole base, each subtending a solitary flower (Figures 2B,C). Production of subtending bracts and flower primordia by main and axillary meristems under long photoperiod growth conditions strongly indicated that the plants were in a reproductive stage (Figures 2B,D,E). It should be noted that during growth under long photoperiod, solitary flowers were observed in the leaf axis of all three cultivars (Figure 2B, Supplementary Figure 1). These flowers reached anthesis under long photoperiod in “NB130” and “NB150” (Supplementary Figure 1). In “NB140,” the solitary flowers were not fully developed and stigmata were not visible (Figure 2B).

### Growth and Development Following Transition to Short Photoperiod

**Figure 3:**
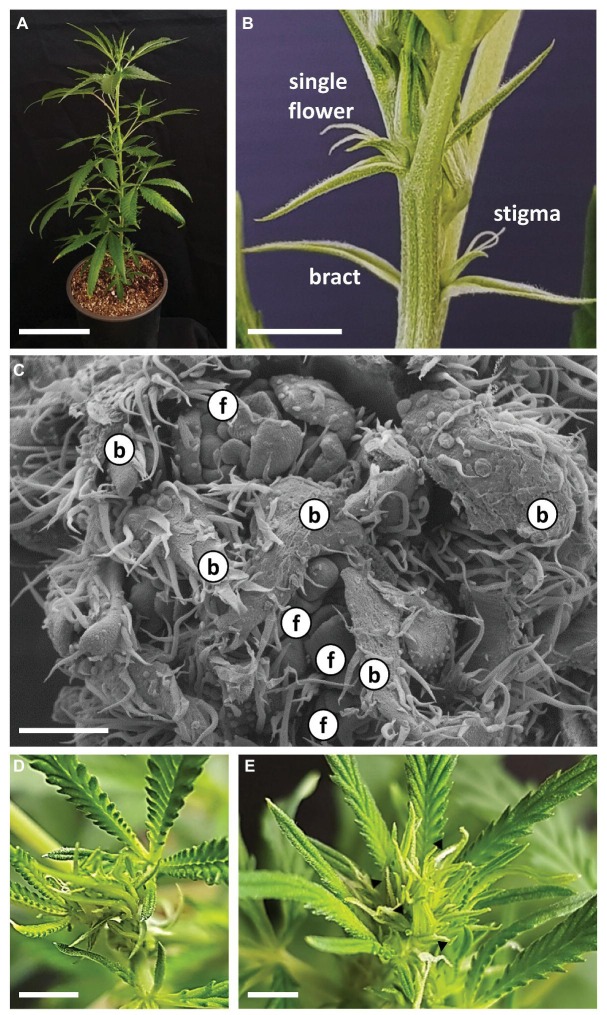
Growth and development of *Cannabis* following transition to short photoperiod conditions. **(A)** Cannabis plant “NB140,” 5 days after transition to short photoperiod conditions. Bar = 10 cm. **(B)** Apical part of main shoot of “NB140,” 5 days after transition to short photoperiod conditions. Bar = 0.5 cm. **(C)** Scanning electron photomicrographs of apical meristem after 7 days of growth under short photoperiod conditions. Bar = 200 μm. **(D)** and (**E)** Shoot apex of “NB140” after 11 and 12 days of growth under short photoperiod conditions. (**E)** Apex phase determined as first day of visible inflorescence. Bars = 0.5 cm. b, bract primordium; f, flower primordium; arrowheads, stigmata.

Three weeks after rooting, young plants were moved to short photoperiod conditions (Figure 3A). After 5 days of short photoperiod, solitary flowers of “NB140” at the leaf axis were fully developed and stigmata were visible (Figure 3B). Since stigmata of solitary flowers in the apical zone might be mistakenly identified as inflorescences, inflorescence flowering was defined as the stage at which at least three pairs of stigmata are visible at the top of the apical shoot. After 8 days of growth under short photoperiod, plants of “NB140” and “NB130” still did not display flowering in the main and lateral inflorescences, whereas “NB150” had already produced visible stigmata at the top of the main shoot. At this point, microscopic analysis of “NB140” meristems revealed intensive branching and primordium differentiation of both vegetative and reproductive organs: bracts and flowers, respectively (Figure 3C). After 11 days under short photoperiod, no stigmata were visible in “NB140” (Figure 3D) or “NB130”, whereas after 12 days of growth, both cultivars developed visible stigmata at the top of the main shoots (Figure 3E). At the same time, apical meristems of the main shoot and lateral branches remained indeterminate and continued producing phytomers, each consisting of a reduced leaf, two bracts, two solitary flowers, and an axillary shoot (Figure 2C).

**Figure 4:**
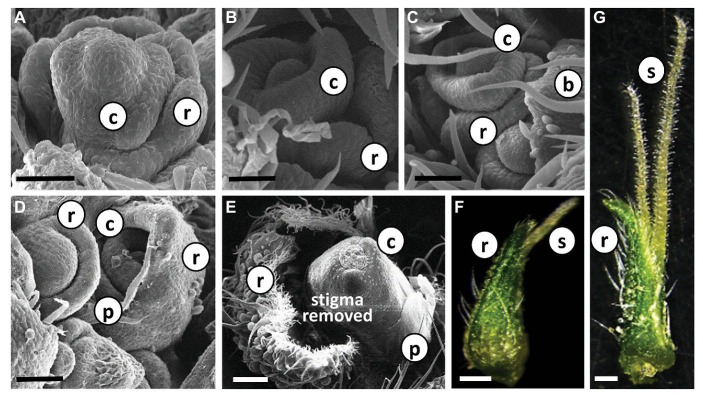
Differentiation and development of individual *Cannabis* flower. **(A)**–(**E)** Scanning electron photomicrographs of consecutive stages of differentiation of female flowers in “NB140.” Bars in **(A–D)** = 50 μm and **(E)** = 200 μm. **(F)** and **(G)** Stereoscope image of developed female flowers with visible glandular trichomes; pre-mature stigmata in **(F)** and fully mature stigmata in **(G)**. Bars = 500 μm. b, bract; r, perigonal bract; c, carpel; p, perianth; s, stigma.

Each individual female flower was located in the axil of a subtending bract that developed at the leaf petiole base (Figure 2B, Supplementary Figure 1). A second type of bract, a perigonal leaf-like bract (= involving bract) that embraced the carpel, and the female flower are differentiated from a common meristem (Figures 4A–D). In addition, a developing perianth was noticeable during early flower differentiation, which later degenerated, lost its identity and looked like a thin membrane (Figures 4D,E). As the flower matured, two stigmata elongated (usually unevenly) and emerged from the perigonal bract. At a later stage, papilla cells developed and covered the stigma from the tip to the basal parts (Figures 4F,G). During flower development, and before stigma elongation, numerous glandular trichomes developed on the perigonal bract that envelops the ovary (Figures 4D–G).

### Plant and Inflorescence Architecture

**Figure 5:**
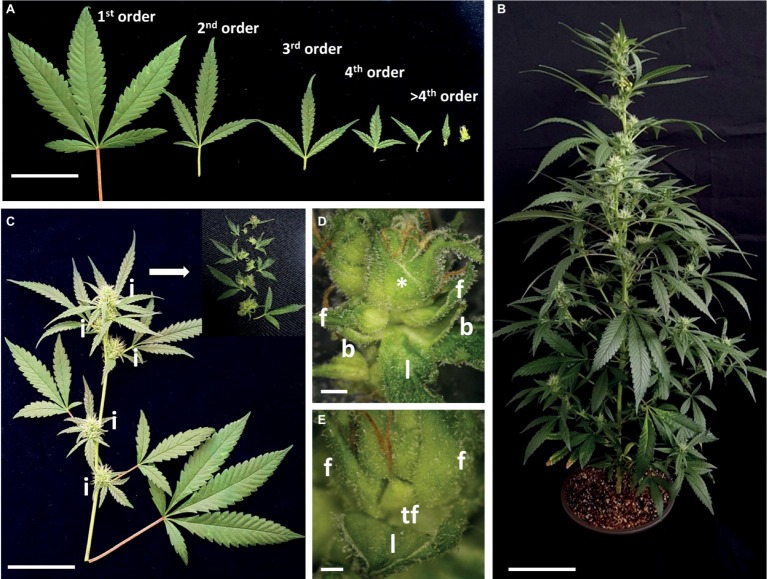
Architecture of *Cannabis* cv. NB140 following inflorescence development. **(A)** Representative image of leaves collected from branches of increasing orders. Bar = 5 cm. **(B)** Flowering cannabis plant “NB140,” 22 days after transition to short photoperiod conditions. Bar = 10 cm. **(C)** Representative image of second-order branch, 22 days after transition to short photoperiod conditions. Insert = disassembled third-order inflorescence. Bar = 5 cm. **(D)** Fifth-order phytomer. Bar = 2 mm. **(E)** Sixth-order phytomer (marked with * in **D**) with terminal flower and two solitary flowers and reduced leaf. Bar = 1 mm. l, reduced leaf; b, bracts; f, solitary flower; i, inflorescence; tf, terminal flower.

Growth and development of the main stem were accompanied by dramatic changes in leaf morphology, with foliage leaves decreasing in size, petiole length, and lobe number (Figures 5A–C). At the full-flowering stage, main inflorescences were noticeable on the apical part of the main, second-, and third-order branches (Figures 5B,C).

**Figure 6:**
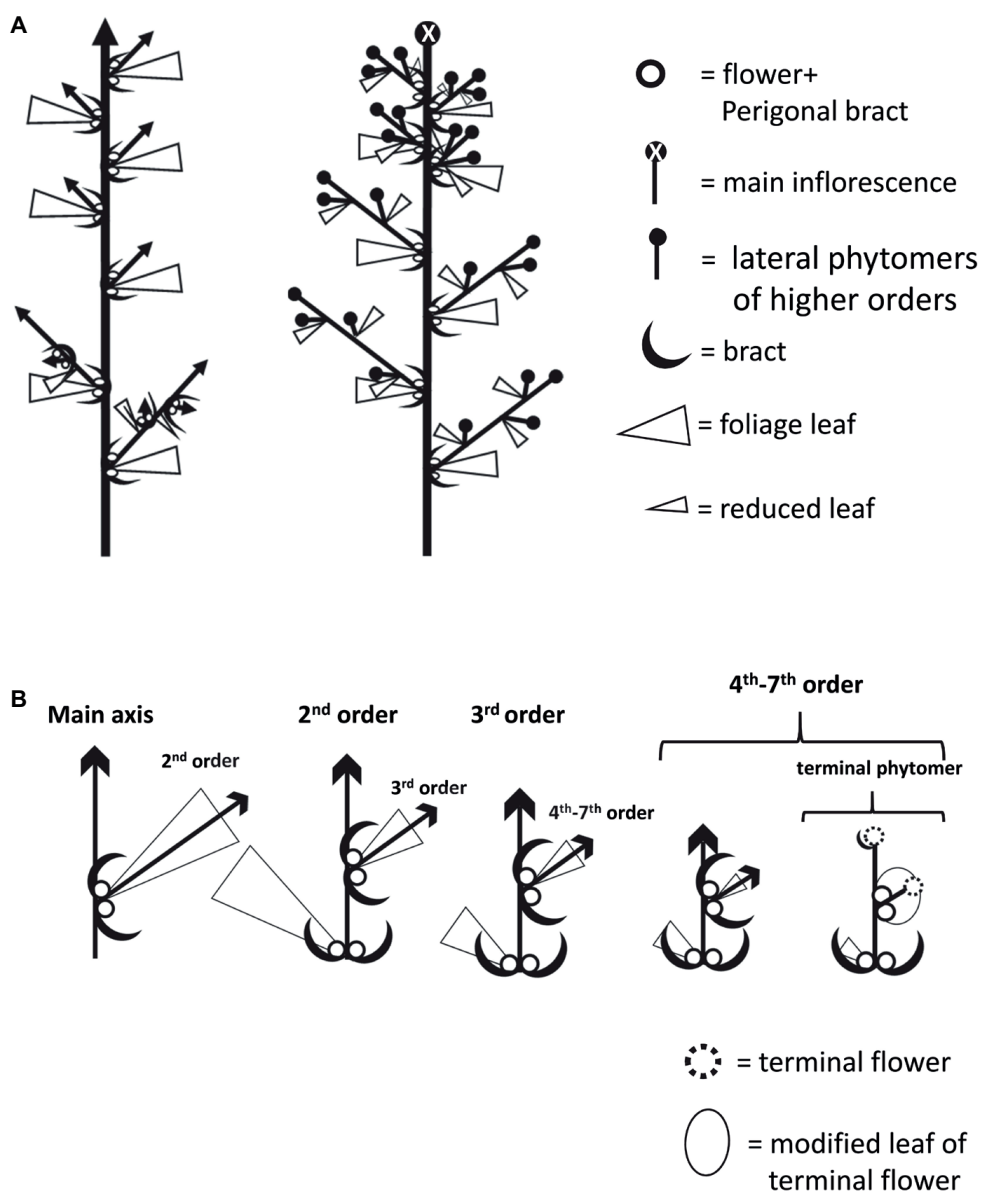
Schematic diagrams of *Cannabis* plant and inflorescence architecture. **(A)** Plant architecture under long photoperiod (left) and short photoperiod (right). **(B)** Architecture of branches and branchlets in increasing order. In terminal phytomer, the leaf is dramatically reduced into a structure that envelops the two solitary flowers and the terminal flowers developed instead of a shoot.

At the microscopic level, each inflorescence was made up of branchlets of higher orders, up to seven visible orders of shoot branching. Each inflorescence phytomer retained the same basic structure as that of plants grown under long photoperiod: two solitary flowers and two bracts located in the base of the reduced leaf petiole and an axillary shoot (Figures 5C,D, 6). The apical meristem then continued the differentiation of new phytomers, while single flowers are differentiated in the axils of the bracts (Figure 5D). Finally, in the terminal sixth- or seventh-order phytomer of “NB150,” the apical meristem terminated by differentiation of a female terminal flower. Therefore, in that cultivar, the terminal phytomer consisted of the last leaf reduced to a scale, embracing the two solitary flowers and the terminal flower (Figures 5E, 6B). Typical traits of the female inflorescence were the high level of dense branching and presence of two single flowers in each of the internodes.

### Variability in Inflorescence Termination

**Figure 7:**
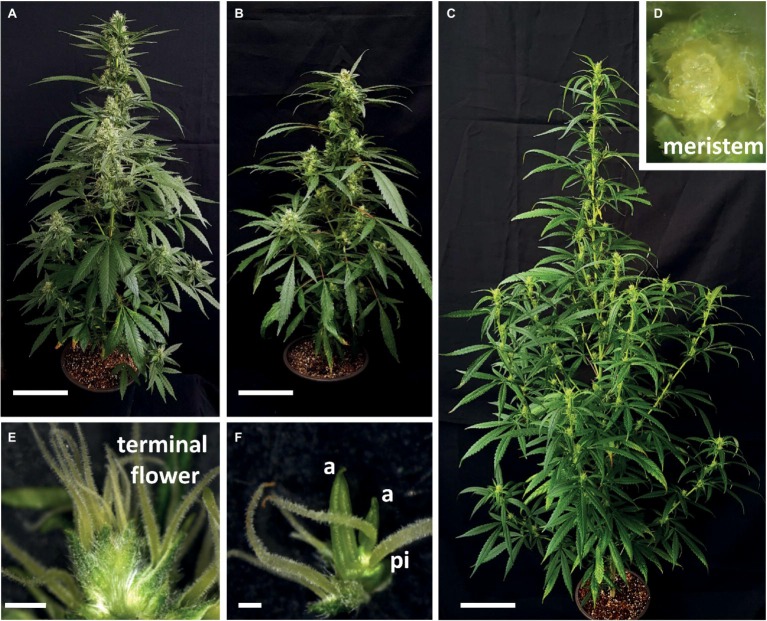
Natural variation in *Cannabis* plant architecture and inflorescence termination. **(A)**–**(C)** Plant architecture of “NB140” **(A)**, “NB150” **(B)**, and “NB130” **(C)**, grown under short day photoperiod for 1 month. Bars = 10 cm. **(D)** Inflorescence meristem of “NB130,” 5 weeks after flowering. **(E)** Terminal flower of “NB140.” Bar = 2 mm. **(F)** Decapitated hermaphrodite terminal flower of “NB150,” both pistils and anthers are differentiated. Bar = 500 μm. a, anther; pi, pistil.

The three studied cultivars differed considerably in plant architecture and inflorescence structure (Figures 7A–C), and termination of the apical meristem occurred in three different setups (Figures 7D–F):

1. After 1 month of cultivation, the main shoot of “NB140” reached 63 ± 4.1 cm in height (Figure 7A), while the longest secondary branches in the lower part of the plant reached 26 ± 6.1 cm. About 8–10 days after visible appearance of the first multiple stigmata at the top of the main inflorescence, the apical meristem terminated by differentiation of the female flower, with normal morphological structure (Figure 7E).
2. The architecture of the “NB150” plants was similar to that of cultivar NB140 (Figure 7B), but the plants were more compact and, after 1 month of cultivation, reached 52.5 ± 5.3 cm in height, with longest secondary branches up to 11.3 ± 2.73 cm. Apical meristems of the female plants ceased their differentiation by production of typical anthers on top of the terminal ovary (Figure 7F). This phenomenon was observed not only in the main apical meristem but also in the most lateral meristems, which terminated their development with hermaphrodite flower formation.
3. Plants of ‘NB130’ had an “open” indeterminate inflorescence. Plants were tall with a loose structure, and after 1 month of cultivation, the main shoot reached 106 ± 3.4 cm in height, with the longest secondary branch up to 42.2 ± 2.7 cm (Figure 7C). Under our experimental conditions, the inflorescence meristem remained indeterminate and continued differentiating even after 7 months (Figure 7D).

## Discussion

*Cannabis* is an annual herb cultivated by humans in almost all parts of the world, from the tropics to alpine foothills. Natural evolution of the species in its centers of origin affected plant physiological requirements for flowering and seed production; as a result, relatively high temperatures and short photoperiod are known to induce and support flowering in cannabis (Cosentino et al., 2012).

### Flower Initiation of Female *Cannabis sativa* Plant Is Day-Neutral

The typical architecture of cannabis plants is a hierarchical branched system (Figures 2, 6). Similar to other dicotyledonous herbs, the adult plant carries numerous repetitive single modular units – phytomers – consisting of an internode and a node (Figure 2; Teichmann and Muhr, 2015). The SAM extends the primary growth axis, while in the leaf axils, lateral meristems differentiate to form morphological structures of higher orders (Figure 2). It is known that in plants originated from seeds and grown under long photoperiod, the vegetative phase ends with differentiation of the first solitary flowers at the fourth to sixth internodes (Cervantes, 2006). Therefore, appearance of these solitary flowers represents the transition from adult vegetative stage to reproductive stage. In horticultural practice, propagation is mainly achieved with cuttings from an adult mother plant. Solitary flowers that have already developed on mother plants, grown under long photoperiod, can persist in the new cuttings that are grown under similar conditions (Figure 2, Supplementary Figure 1). Cannabis producers and researchers consider long photoperiod to be “non-inductive” or “vegetative” growth conditions, but the development of solitary flowers clearly indicates that the plant at this stage cannot be defined as vegetative or non-inductive in the classical sense (Figure 2). Therefore, flower induction of solitary flowers is probably age-dependent and is controlled by internal signals, but not by photoperiod.

In the model plant *Arabidopsis thaliana*, which is a facultative long-day flowering plant, more than five flowering pathways have been defined, including environmental, autonomous, age-dependent, and gibberellin pathways (Cho et al., 2017). In day-neutral flowering plants, such as tomato, flower induction is mainly governed by age-dependent and gibberellin pathways (Silva et al., 2018). As regards the development of solitary flowers in *Cannabis*, in all studied cultivars, flowers are differentiated under both long and short photoperiods (Figures 2, 3, Supplementary Figure 1). Therefore, from a flower-induction standpoint, the plant can be seen as day-neutral.

Following flower induction, solitary flowers of “NB150” and “NB130” reached anthesis under both short and long photoperiod growth conditions, whereas in “NB140,” short photoperiod was required for post-induction flower bud maturation and anthesis (Figures 2, 3, Supplementary Figure 1). Similarly, *Caryopteris* and *Passiflora edulis* have no photoperiod requirements for flower induction but require a specific photoperiod length for flower maturation: in *Caryopteris* flowers, initiation does not have photoperiod requirements, but anthesis only occurs at day length shorter than 16 h (Piringer et al., 1963); in *P. edulis*, flower induction is independent of environmental cues, and long photoperiod is required for the flower to complete its development (Nave et al., 2010). Isolation and characterization of the genetic and physiological elements involved in photoperiodic development of solitary flowers will be useful for better understanding the differences between *Cannabis* cultivars of different origins.

Photoperiod has a wide-ranging effect on plant development, e.g., controlling flowering time, meristem termination, bud dormancy, and branching. In wheat, onion, rice, and other crops, photoperiod triggers the initial elongation of flower stalks and flower initiation (Blümel et al., 2015). Photoperiod, like other environmental stimuli, regulates plant responses through internal signals that affect plant architecture. In *Arabidopsis*, the florigen genes *FLOWERING LOCUS T* (*FT*) and *TWIN SISTER OF FT* (*TSF*) play dominant roles in the promotion of lateral shoot development independently of their effect on the floral transition (Hiraoka et al., 2013). In addition, BRANCHED1/TEOSINTE BRANCHED1-LIKE 1 transcription factor, a key negative regulator of branching in *Arabidopsis* that belongs to the TEOSINTE BRANCHED1, CYCLOIDEA, and PCF family, can interact within axillary meristems with both FT and TSF and inhibit their functions (Niwa et al., 2013). We argue that in *Cannabis*, a short photoperiod orchestrates intense branching of the inflorescence, with floral initiation that occurs independently of short photoperiod.

### The Inflorescence of *Cannabis* Is a Highly Branched Compound Raceme

When cannabis plants were moved to a short photoperiod, compressed inflorescences developed at the top of the main stem and second- and third-order branches (Figures 3, 5, 6). Each inflorescence consisted of condensed higher-order branchlets. Each condensed branchlet retained the same phytomer structure as that of the larger phytomers developed under long day and consisted of reduced leaves, bracts, one or two solitary flowers, and an axillary shoot (Figures 5, 6). Similarly, the structure of the female cannabis inflorescence was described more than 100 years ago as “pistillate flowers…developed two by two in the axils of leaves representing the first small branchlets of the secondary axillary branch which develops between them” (Reed, 1914).

The *Cannabis* inflorescence can be defined as a highly branched compound raceme. It is characterized by monopodial growth, with persistent apical meristem and axillary indeterminate inflorescences of higher orders (Figure 6). The development of the inflorescence is acropetal and lateral racemes are produced prior to terminal flower differentiation. In most cases, open inflorescences – such as racemes and compound racemes – do not produce terminal flowers, as in *Arabidopsis*, *Antirrhinum*, and *Cannabis* “NB130” (Figure 7; Claßen-Bockhoff and Bull-Hereñu, 2013). However, in some racemes, terminal flowers appear naturally, as in *Digitalis purpurea* (Claßen-Bockhoff and Bull-Hereñu, 2013) or *Cannabis* “NB150” and “NB140” (Figure 7). Differentiation of terminal flowers of racemes can be caused by mutations in the genes regulating meristematic identity (Lifschitz et al., 2014; Park et al., 2014).

Under our experimental conditions, the apical meristems of the studied cultivars demonstrated different paths of cessation of inflorescence differentiation: the indeterminate meristem of “NB130,” meristem termination with an apical female flower in “NB140,” and a malformed stamenoid-pistillate flower in “NB150.” Sex in *Cannabis* is governed by heteromorphic chromosomes (Hall et al., 2012). Yet, sex reversal in cannabis involves ethylene and gibberellin signaling (Sarath and Mohan Ram, 1979). It may therefore be that masculinization of the terminal flower in “NB150” was caused by stress or by other ethylene- or gibberellin-related signals.

Further research should examine the genetic regulation of the interplay between flower initiation and branching in the *Cannabis* inflorescence. Considering that the trichomes are located mainly on vegetative parts of the inflorescence (Andre et al., 2016; Raman et al., 2017), that intense branching leads to internode reduction, and that there is differentiation of a compact inflorescence with numerous bracts, an understanding of the genetic mechanism governing branching and florogenesis will lay the foundation for genetic, biotechnological, and physiological applications to modify architecture and to maximize plant productivity and uniformity in medical *Cannabis*.

## Data Availability

All datasets generated for this study are included in the manuscript and/or the Supplementary Files.

## Author Contributions

NB, RK, and BS-R planned the experiments. BS-R supervised the project and, together with SD, carried out the experiments. BS-R and RK wrote the manuscript with support from NB. SD contributed to the final version of the manuscript.

### Conflict of Interest Statement

The authors declare that the research was conducted in the absence of any commercial or financial relationships that could be construed as a potential conflict of interest.

## Footnotes

^1^https://www.cannabisbusinesstimes.com/article/the-nomenclature-of-female-flowers/
